# Adsorption and mitigation impact of the monosodium glutamate (C_5_H_8_NO_4_Na) bio-molecules on the steel rebar corrosion in the chloride-contaminated simulated concrete pore solution

**DOI:** 10.1038/s41598-023-38111-y

**Published:** 2023-07-10

**Authors:** Sahel Mohammadkhah, Ali Dehghani, Bahram Ramezanzadeh

**Affiliations:** 1 Department of Chemical and Biomolecular Engineering, North Carolina State University, P.O. Box 27606, Raleigh, NC USA; 2 Department of Surface Coatings and Corrosion, Institute for Color Science and Technology, P.O. Box 16765-654, Tehran, Iran

**Keywords:** Biomaterials, Techniques and instrumentation

## Abstract

Corrosion has caused significant annual costs for building construction and civil architectural designs. In this study, Monosodium glutamate (GLU) was proposed as a potential candidate for long-lasting corrosion inhibition to slow down the rate of corrosion in the concrete pore environment. In this regard, the electrochemical and morphological properties of the various GLU concentrated systems between 1 to 5 wt% in the simulated concrete pore solution media were investigated. According to the EIS results, adding 4 wt% of GLU could reduce the mild steel corrosion process by 86% through a mixed inhibition mechanism. Also, the polarization records represented that the samples’ corrosion current density was diminished to 0.169 µA cm^−2^ after the addition of 4 wt% GLU into the harsh environment. Using the FE-SEM method, the growth of the GLU layer over the metal substrate was demonstrated. The results of spectroscopic methods, i.e., Raman and GIXRD, demonstrated that GLU molecules were successfully adsorbed over the surface of the metal. Contact angle test outcomes showed that by increasing the GLU concentration to its optimum level (4 wt%), the surface hydrophobicity was dramatically raised to 62°.

## Introduction

The combination of metals with cement as a reinforcement concrete is widely utilized in civil engineering and design for better mechanical characteristics and longevity of structures^1,2^. However, the corrosion of applied metallic materials and steel bars especially in marine environments and chloride-contaminated media is one of the major causes of degradation in modern structures^3,4^. Due to metal corrosion, a huge amount of resources are being spent annually for repetitive repair and maintenance of structures. While the corrosion process cannot be stopped completely, it can be drastically slowed down. Various approaches have been suggested to improve the anticorrosion features of reinforcing metallic materials^4,5^. Among all proposed protection methods, corrosion inhibitors have gained a lot of attention in recent studies^1–3,5–9^.

Corrosion inhibitors are divided into two primary categories: organic and inorganic. Due to their cost and ease of use in various industries, organic inhibitors are chosen over inorganic ones for use against the corrosion process^4^. The inhibition mechanism of organic inhibitors is based on electron donation^6–9^. Evidently, organic inhibitors have occupied a large number of heteroatoms. These atoms are responsible for the chelation of inhibitive organic agents with the steel surface to extend the organic protective film^6–9^. Taking all the advantages of organic inhibitors into account, numerous studies have examined their effects on the corrosion prevention of metallic samples in the presence of chloride-contaminated simulated concrete pore solutions (CPS).

For instance, the research carried out by Wang and Zou showed that Calcium lignosulfonate can be used as a potent inhibitor to lessen the corrosion of carbon steel in CPS. Electohcmeial data evidenced that the addition of 0.008 mol L^−1^ Calcium lignosulfonate can attain about 93% of corrosion protection after 720 h exposure to the corrosive media^10^. Polycarboxylate polymers were considered another reliable inhibitor by the Fazayal research team. This work concentrated on comparing different functionalized Polycarboxylate polymers and various environmental factors to optimize the inhibitor efficiency. Overall records declared that the utilized inhibitor substantially reduced the pitting corrosion in the concrete pore media^11^. Jiang’s assessments clarified that Deoxyribonucleic acid was another potential inhibitor for reinforcing the anticorrosion index of steel against concrete pore media. This study analyzed the effect of inhibitor and chloride ions concentration on steel corrosion. Results have demonstrated that even the low concertation of Deoxyribonucleic acid can provide an excellent corrosion protection degree. The research showed that the inhibition index can be tuned by increasing the inhibitor concentration. On the contrary, chloride ions are responsible for severe corrosion, and the enhancement of chloride ions can impose higher aggression^12^.

Monosodium glutamate is one of the important organic inhibitors^13,14^. It can be transformed into ionic forms in harsh corrosive solutions and protect metal via various mechanisms^13,14^. In neutral conditions, they can be chemically adsorbed by an electron donation mechanism, while the ionic form of GLUs can make the steel surface passive through physical adsorption^13,14^. Recently, GLUs have been widely applied as organic inhibitors in different harsh corrosive systems^13,14^.

The present work focused on introducing GLU as a substantial corrosion inhibitor for reinforcing the anti-corrosion performance of steel substrate against chloride-contaminated CPS. First, polarization and EIS techniques were used to explore GLU anti-corrosion efficiency. Next, via the FE-SEM and contact angle tests the steel surface nature was analyzed. Eventually, the results of spectroscopic GXRD and Raman technics have demonstrated the steel surface chemistry in detail.

## Experimental

### Materials and methods

In order to prepare the CPS, the commercial NaOH, KOH, and Ca(OH)_2_ were purchased from Merck Co., and the salty environment was generated by pure NaCl powder prepared from Mojalali Co. In the present study, GLU was purchased by Merck Co. and used as a potential inhibitor for increasing the St-12 samples' corrosion resistance in a chloride-contaminated CPS. The St-12 sheets were supplied by Mobarakeh Co. with a chemical composition of 0.10 wt% C, 0.035 wt% P, 0.45 wt% Mn, 0.035 wt% S, 0.007 wt% N, and the rest of Fe.

### Samples and electrolytes

All of the experiments were conducted over the St-12 samples. Prior to applying each analysis, samples were precisely sanded with 180, 420, 640, and 1200 carbide papers and then deoxidized/degreased with acetone and double distilled water.

In order to make the CPS, the 3.5 wt% NaCl solution was prepared and then 4 g/L NaOH, 1.6 g/L Ca(OH)_2_, 7.2 g/L KOH and 0.5 g/L CaSO_4_ were added immediately^15–18^. The final mixture pH was set at 12.5–13 and the solution was selected as a reference electrolyte. Thereafter, the reference electrolyte was reinforced by adding 1, 2, 3, and 4 wt% GLU.

### Electrochemical investigations

The protection mechanism was estimated by a polarization test. The polarization curves were recorded after 2 days of exposure to the electrolytes and electrochemical parameters were extracted by the IviumSoft program. Moreover, the GLU effects over the anticorrosion index of steel substrate were estimated by the EIS test. The results were reported after 1, 3, 5, 24, and 48 h subjection times. The electrochemical parameters were achieved by the ZSimp program and the deviations were computed either. The electrochemical setup was prepared by a normal three-electrode system including reference (Ag.AgCl), counter (platinum), and work (steel) electrodes. In this work, the polarization was carried out in − 250 mV until + 250 mV range with a 1 mV s^−1^ scanning rate. Besides, the EIS frequency is limited to 0.01–10,000 Hz with ± 0.01 V perturbation.

### Surface analysis

The sample morphologies were deeply investigated by FE-SEM (Hitachi S-4700) equipped with EDS/mapping for elemental evaluation of the film formed on the metal surface. For further analysis, the surface topography was studied by AFM (Park XE-7). Since surface hydrophobicity is a reliable approach for predicting the surface nature, the contact angle test was operated over all immersed samples (homemade device). Eventually, for evidencing the successful adsorption of GLU, the surface chemistry was analyzed by FT-IR (Alpha II), GXRD (Bruker D8), and Raman (RMP-500) spectroscopies.

### Protection method

As much literature evidenced, a passive layer can be easily developed over the steel substrates. Based on literature and previous studies, in the alkaline media, the steel is protected through the development of magnetite (Fe_3_O_4_) passive film over the metallic substrate. Mainly, magnetite is formed via the oxidation of iron(II) hydroxide which is presented in Eq. (1)^19^.

1 $${\text{3 Fe}}\left( {{\text{OH}}} \right)_{{2}} + {\text{ 2OH}}^{ - } \to {\text{Fe}}_{{3}} {\text{O}}_{{4}} + {\text{ 4H}}_{{2}} {\text{O }} + {\text{ 2e}}^{ - }$$

However, the pre-developed layer is not intact and highly dense enough. As a result, the low integrate sides will behave as anodic zones, whereas the undamaged sites can behave as cathodic regions. In these conditions, the electrochemical reactions will occur as follows^20^:

2 $${\text{Fe }} \to {\text{ Fe}}^{{2 + }} \, + {\text{ 2e}}^{ - } \;\;\;\;\left( {\text{anodic sides}} \right)$$

3 $$\frac{1}{2}{\text{O}}_{{2}} \, + {\text{ H}}_{{2}} {\text{O }} + {\text{ 2e}}^{ - } \, \to {\text{2(OH)}}^{ - } \;\;\left( {\text{cathodic sides}} \right)$$

4 $${\text{Fe}}^{{{2} + }} \, + {\text{ 2OH}}^{ - } \, \to {\text{Fe}}({\text{OH}})_{{2}} \to {\text{Fe}}({\text{OH}})_{3} \;\;\left( {\text{corrosion products}} \right)$$

After adding the GLU to the CPS, the GLUs molecules can participate in the chloride-based attacks. The free chloride ions in CPS can attack the pre-constructed passive film over the steel surface that leaves metal pitting, resulting in the corrosion products construction^21^. In the absence of GLU, the chloride ions have a high probability of reacting with the Fe^2+^ and Fe^3+^ cations to construct FeCl salts. However, the charged GLUs can easily interact with the Fe ions and forms the Fe(GLU) layer^22,23^.

5 $${\text{Fe}}^{{{3} + }} \, + {\text{ 3GLU}}^{ - } \, \to {\text{Fe(GLU)}}_{{3}}$$

Additionally, the GLU can interact with the charged Ca cations, making a physical barrier over the immersed substrate^24^:

6 $${\text{Ca}}^{{{2} + }} \, + {\text{ 2GLU}}^{ - } \to {\text{Ca(GLU)}}_{{3}}$$

In addition to the physical reactions, GLU can interact through chemical interactions. The presence of C=O groups is a reliable site for electron donation with free orbitals of iron. The chemical chelation between the GLUs and Fe ions results in the anodic protection of metal against corrosion.

## Results and discussion

### Protection mechanism analysis (polarization studies)

**Figure 1:**
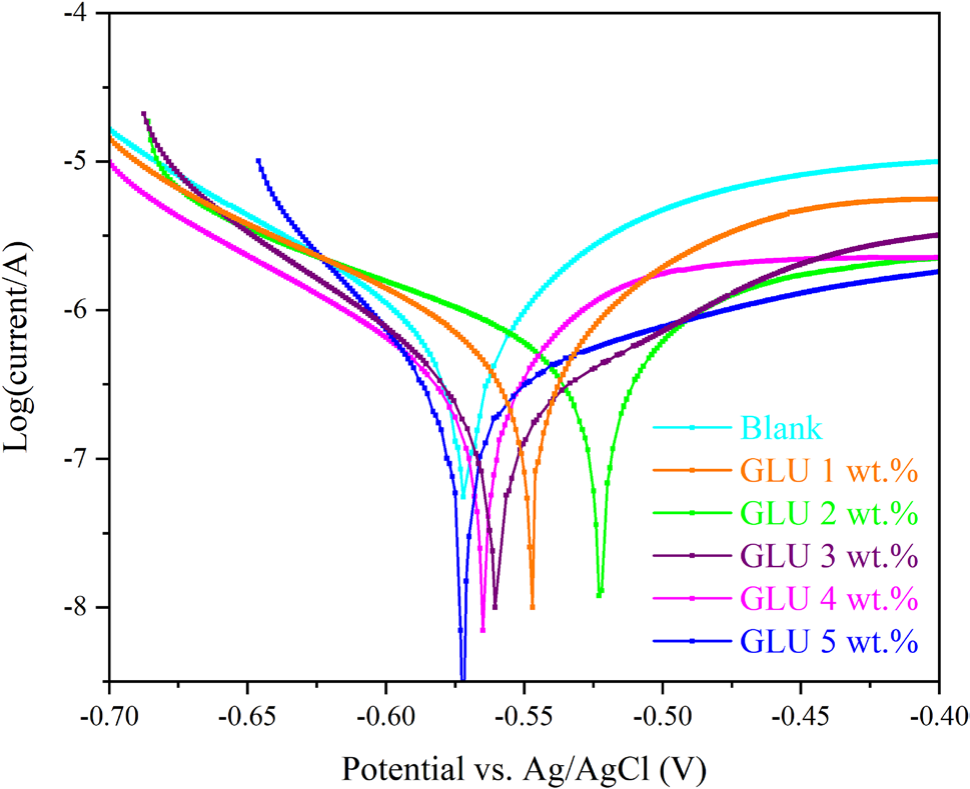
The polarization plots after 48 h steel exposure to the CPS solution with different concentrations of GLU.

In order to explore the GLU protection mechanism, the polarization curves were presented (Fig. 1), and factors were listed in Table 1. Results displayed that the blank sample provided the highest corrosion current density (*i*_corr_), confirming the high aggression rate of steel in simulated concrete media. Meanwhile, by enhancing the GLU concertation in the harsh environment, the *i*_corr_ descended continuously until reaching 0.169 µA cm^−2^ from the 4 wt% GLU protected sample. Results showed the GLU effectiveness in steel corrosion decrement and by increasing the GLU concertation up to 4 wt%, its anti-corrosion performance was enhanced, and over that the corrosion inhibition efficiency was diminished^25,26^. Records from corrosion potential (*E*_corr_) and anodic branch variations reflected that GLU has mixed protection potential. The changes over both cathodic and anodic slopes, “*β*c and *β*a”, are attributed to the GLU adsorption over the anodic sites and GLUs chelation with oxidized particles over the cathodic sites of metal^27^.

**Table 1:** Data originated from the polarization test.

	Blank	GlU 2%	GlU 3%	GlU 4%	GlU 5%
*i*_corr_ (μA cm^−2^)	0.861	0.258	0.253	0.169	0.172
*E*_corr_ (V)	− 0.545	− 0.551	− 0.541	− 0.519	− 0.577
β_a_ (V dec^−1^)	0.058	0.098	0.099	0.121	0.085
β_c_ (V dec^−1^)	− 0.105	− 0.124	− 0.129	− 0.134	− 0.043
*η* (%)	–	70.0	70.6	80.3	80.0

### EIS explorations

**Figure 2:**
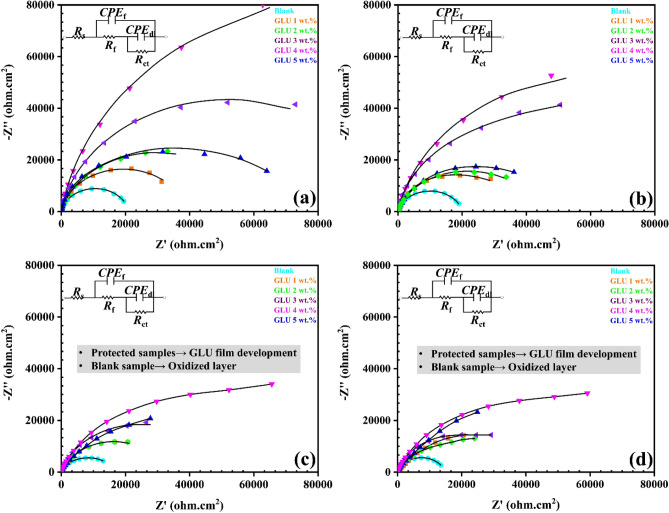
The Nyquist plots from the protected and unprotected CPS after (**a**) 1 h, (**b**) 5 h, (**c**) 24 h, and (**d**) 48 h exposure.

**Figure 3:**
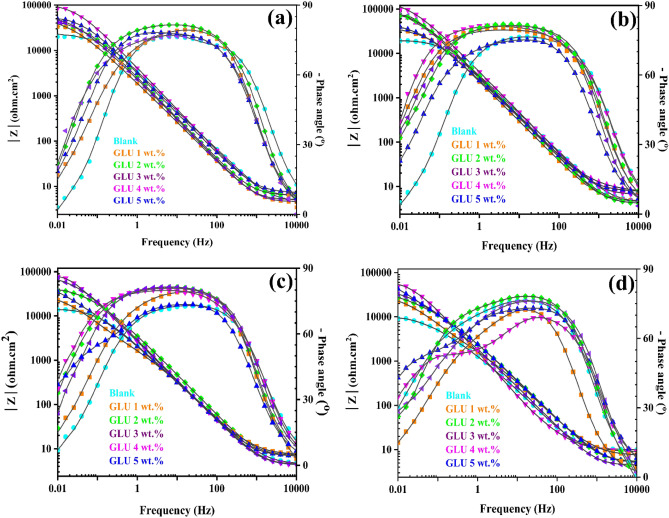
The Bode plots from the protected and unprotected CPS after (**a**) 1 h, (**b**) 5 h, (**c**) 24 h, and (**d**) 48 h exposure.

After exploring the GLU protection mechanism via polarization test, the EIS test was conducted to evaluate the GLU inhibition index as a function of immersion time (Figs. 2 and 3). Due to the steel substrate coverage with GLU protective layer, all of the protected samples’ plots were fitted with a two-time constant model and the development of an oxidized passive layer over the blank sample is responsible for the generation of a new time constant related to the inhibitor film (Table 2).

**Table 2:** Extracted electrochemical variables from the EIS records.

Particles type, immersion time (hours)	*R*_*c*_ (Ω cm^2^)	*CPE*_*c*_		*R*_*ct*_ (Ω cm^2^)	*CPE*_*dl*_		*R*_*t*_ (Ω cm^2^)	|*Z*|_0.01 Hz_ (Ω cm^2^)
		*Y*_0,c_ (μΩ^−1^ cm^−2^ s^n^)	*n*_*c*_		*Y*_0,dl_ (μΩ^−1^ cm^−2^ s^n^)	*n*_*dl*_		
Blank, 1	547.1	45.1	0.87	21,720	9.5	0.87	22,267.1	20,154
Blank, 5	521.1	48.4	0.82	14,329	11.9	0.83	20,850.1	19,421
Blank, 24	315.5	59.1	0.76	16,534	16.2	0.75	16,849.5	15,001
Blank, 48	311.2	70.5	0.72	12,835	19.6	0.69	13,146.2	9246
Glutamate 1 wt%, 1	912.3	39.4	0.85	43,912	6.4	0.98	44,824.3	31,056
Glutamate 1 wt%, 5	421.5	41.1	0.81	37,416	7.1	0.97	42,367.2	31,365
Glutamate 1 wt%, 24	389.0	42.3	0.81	34,015	8.7	0.95	34,404.0	21,866
Glutamate 1 wt%, 48	345.1	45.7	0.79	39,410	9.9	0.93	39,755.1	21,225
Glutamate 2 wt%, 1	1142.1	25.1	0.88	79,241	6.4	0.98	80,383.1	33,297
Glutamate 2 wt%, 5	1005.9	28.4	0.87	53,278	6.9	0.97	54,283.9	70,021
Glutamate 2 wt%, 24	952.1	30.6	0.81	33,790	7.1	0.95	34,742.1	36,522
Glutamate 2 wt%, 48	891.8	39.8	0.79	40,016	7.5	0.94	40,907.8	28,659
Glutamate 3 wt%, 1	2477.0	21.8	0.91	120,415	6.6	0.97	122,892.0	43,600
Glutamate 3 wt%, 5	2337.9	21.9	0.89	92,642	6.8	0.94	94,979.9	72,315
Glutamate 3 wt%, 24	2289.5	31.3	0.87	50,877	6.8	0.91	53,166.5	70,355
Glutamate 3 wt%, 48	2199.0	33.5	0.83	53,189	7.0	0.91	55,388.0	32,400
Glutamate 4 wt%, 1	1917.3	22.5	0.89	158,914	6.4	0.93	160,831.3	84,570
Glutamate 4 wt%, 5	1889.2	25.1	0.88	144,832	6.6	0.89	146,721.2	92,164
Glutamate 4 wt%, 24	1805.2	29.6	0.80	103,160	7.1	0.87	104,965.2	83,410
Glutamate 4 wt%, 48	1751.9	37.7	0.79	99,661	7.3	0.82	101,412.9	52,189
Glutamate 5 wt%, 1	1691.8	23.7	0.89	96,195	6.5	0.98	97,886.8	49,650
Glutamate 5 wt%, 5	1554.4	25.7	0.88	69,487	6.9	0.96	71,041.1	39,270
Glutamate 5 wt%, 24	1502.79	30.9	0.83	40,826	7.0	0.93	42,328.6	34,720
Glutamate 5 wt%, 48	1430.059	37.2	0.81	45,440	7.3	0.93	46,870.2	34,040

Results from the blank sample confirmed that the maximum total resistance (*R*_t_ = *R*_f_ + *R*_ct_ + *R*_s_) recorded for the bare sample was 20,154 Ω cm^2^ only. The high value of total resistance in the initial times corresponds to the oxidized layer development over the steel substrate that resulted from the corrosive reactions. Although the Fe-based oxide particles can provide slight protection over the cathodic sites, their instability is a big issue. The Fe oxides can be easily detached from the steel surface and only short-term protection can be observed^28^. However, in the long term, the iron oxide film detachment causes steel corrosion continuation and notable degradation^29^. Explorations from the 1 wt% GLU-protected sample has proved that the GLU acted as a proper active inhibitor for the simulated concrete solution aggressiveness control. Final records from EIS results demonstrated that the highest resistance was achieved from the 4 wt% protected sample. Apparently, by extending the immersion time, the GLU-protected sample's film resistance (*R*_f_) endured slight enhancement. The increments are totally originateed from the GLU molecules' successful adsorption and the protective layer construction^30^. By increasing the GLU concentration to 2 wt% and 3 wt%, remarkable improvements can be observed owing to the higher steel surface coverage in the higher concentrations. In other words, the inhibition proficiency of the developed layer was improved by increasing the GLUs concentration. Achievements proved that the “*R*_f_” of the 2 wt% GLU protected sample ranged between 891 and 1141 Ω cm^2^, whereas it was enhanced until 2537 Ω cm^2^ for the 3% wt% GLU. Clearly, the highest protection index was achieved from the 4 wt% GLU concentration. Thanks to noticeable GLU adsorption on the metal surface, the “*R*_f_” of 4 wt% GLU protected sample reached 3889 Ω cm^2^, and “*R*_t_” provided around 94,164 Ω cm^2^ meanwhile. However, by addition of 5 wt% GLU, it is obvious that the resistance of the system slightly dropped. This alteration could be explained based on the proper dispersion of the GLU that leads to insufficient inhibition performance. All that being said, the 4 wt% concentration can be highlighted as proper concertation for achieving the highest anticorrosion index from the designed system.

For the analysis of the anticorrosion index of the protected samples, the inhibition efficiency was computed^28,31^. Computations clarified that 4 wt% GLU could prohibit 87% of the chloride-contaminated CPS corrosiveness, whereas the 5 wt%, 3 wt%, 2 wt%, and 1 wt% purchased around 80%, 82%, 72%, and 50% protection indexes, respectively.

### Inhibition mechanism

Upon the subjection of the steel sample to the CPS, cathodic and anodic reactions will initiate that is responsible for the construction of corrosion products. Among all types of corrosion products, magnetite particles construction is far more usual^16^:

7 $${\text{3Fe(OH)}}_{{2}} {\text{ + 2OH}}^{ - } \to {\text{Fe}}_{{3}} {\text{O}}_{{4}} {\text{ + 4H}}_{{2}} {\text{O + 2e}}^{ - }$$

Meanwhile, the redox reactions involve the construction of Fe (III) oxides and FeOOH compounds. The conversion of Fe(III) oxide to Fe(II) oxide during oxidation/dehydration will occur as follows^16^:

8 $${\text{Fe}}_{{3}} {\text{O}}_{{4}} {\text{ + 2OH}}^{ - } \to {3}\alpha{\text{-Fe}}_{{2}} {\text{O}}_{{3}} {/}\gamma{\text{-Fe}}_{{2}} {\text{O}}_{{3}} {\text{ + H}}_{{2}} {\text{O + 2e}}^{ - }$$

Simultaneously, the Fe_3_O_4_ are crucial compounds for the development of FeOOH via oxidation/dehydration mechanism^16^:

9 $${\text{2Fe}}_{{3}} {\text{O}}_{{4}} {\text{ + 2OH}}^{ - } + {\text{H}}_{{2}} {\text{O}} \to {3}\alpha{\text{-FeOOH/}}\gamma{\text{-FeOOH + e}}^{ - }$$

Furthermore, since the harsh media has been saturated with carbonate ions, the Fe carbonates can be generated by a one-step process:

10 $${\text{Fe}}^{{2 + }} {\text{ + CO}}_{{3}}^{2 - } \to {\text{FeCO}}_{{3}}$$

According to the explanations, the subjected sample will govern by a passive layer that consists of FeOOH, Fe_2_O_3_, and Fe_3_O_4_ phases. However, the passive layer will suffer pitting corrosion as chloride ions are still available in free forms in concrete pore media^32^. Owing to the positive charge of the metal surface, Cl^−^ ions tend to be physically adsorbed over the surfaces. In these circumstances, the passive layer will destruct due to the penetration of Cl^−^ ions and pH drop^33^.

**Figure 4:**
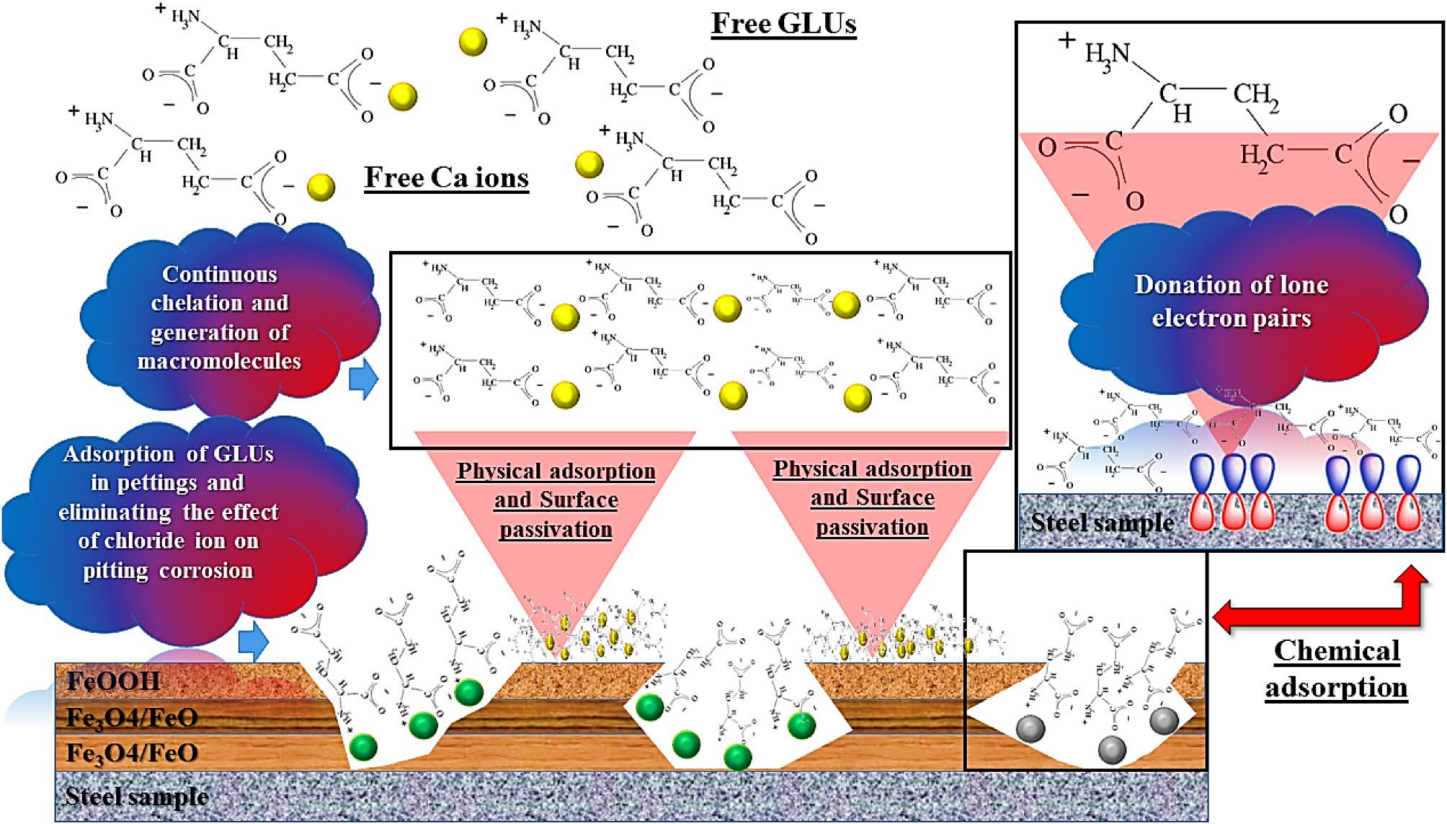
Protection mechanism schematic of GLUs in a corrosive CPS environment.

However, after the inclusion of glutamate molecules, these particles can hinder further aggression by following different strategies. As expressed in polarization studies, GLU can retard the aggression extent by suppressing both anodic and cathodic reactions. (i) Since the GLUs are available in ionic forms (positive charge near N element and negative charge near O element), they have a competition with chloride ions for adsorption over the steel surface. (ii) The pit zone is a potential side for the development of steel aggression, but by the migration of GLUs in these zones, the corrosion acceleration will limit prosperously. (iii) Also, GLUs can neutralize the acidic atmosphere of pit sides which reduces the corrosion index. (iv) Apparently, the GLUs has occupied with heteroatoms. By donation of lone electron pairs with empty orbitals of Fe, a stable film will be formed that promotes the inhibition index. (v) Simultaneously, a synergistic effect can be observed. The free GLU molecules can react with Ca cations and continuous reactions can generate a heavy complex. Upon adsorption, the surface will be passivated by a GLU:Ca physical barrier (Fig. 4).

### Elemental records and surface morphology

**Figure 5:**
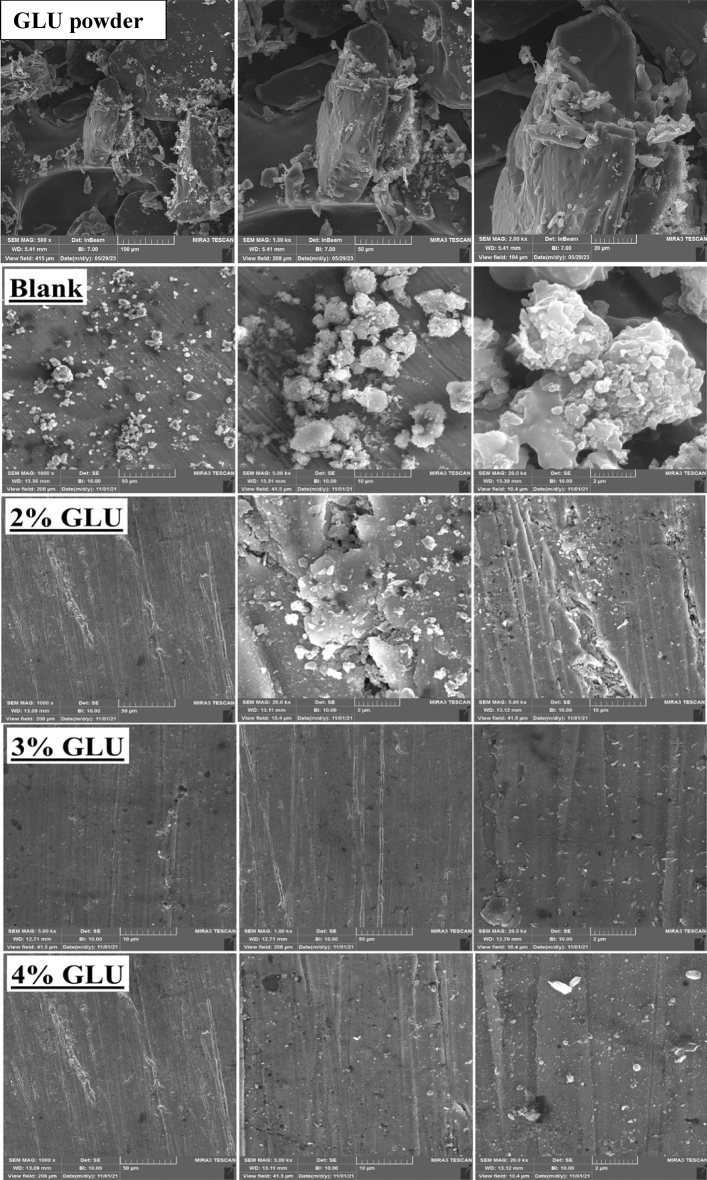
FE-SEM captures from the protected and unprotected samples after 48 h immersion in CPS.

Figure 5 shows the SEM images of the GLU powder as well as the protected metal surface. The morphological captures from the samples' surfaces after 48 h of immersion have demonstrated that the bare sample was remarkably corroded and the corrosion products totally covered the substrate (Fig. 5). However, by introducing the GLUs into the concrete simulated electrolyte, a highly dense protective film covered the surface. The surface coverage by GLU film can limit steel accessibility from the harsh media, leading to steel aggression mitigation. Clear observations from the micrographs evidenced that some porosities still existed over the developed layer even after steel coverage by GLU film. The presence of porosities can leave catastrophic effects on the protective film performance in the long term. In detail, these lacks and imperfections over the inhibitive film enhance the electrolyte permeation into the layer that brings interior pressure, layer destruction, and performance mitigation. Apparently, the number of porosities declined by raising the GLU concentration. The disappearance of these micro-porosities can prosperously control the solution diffusion in high volumes and higher protection index will be achieved consequently.

**Figure 6:**
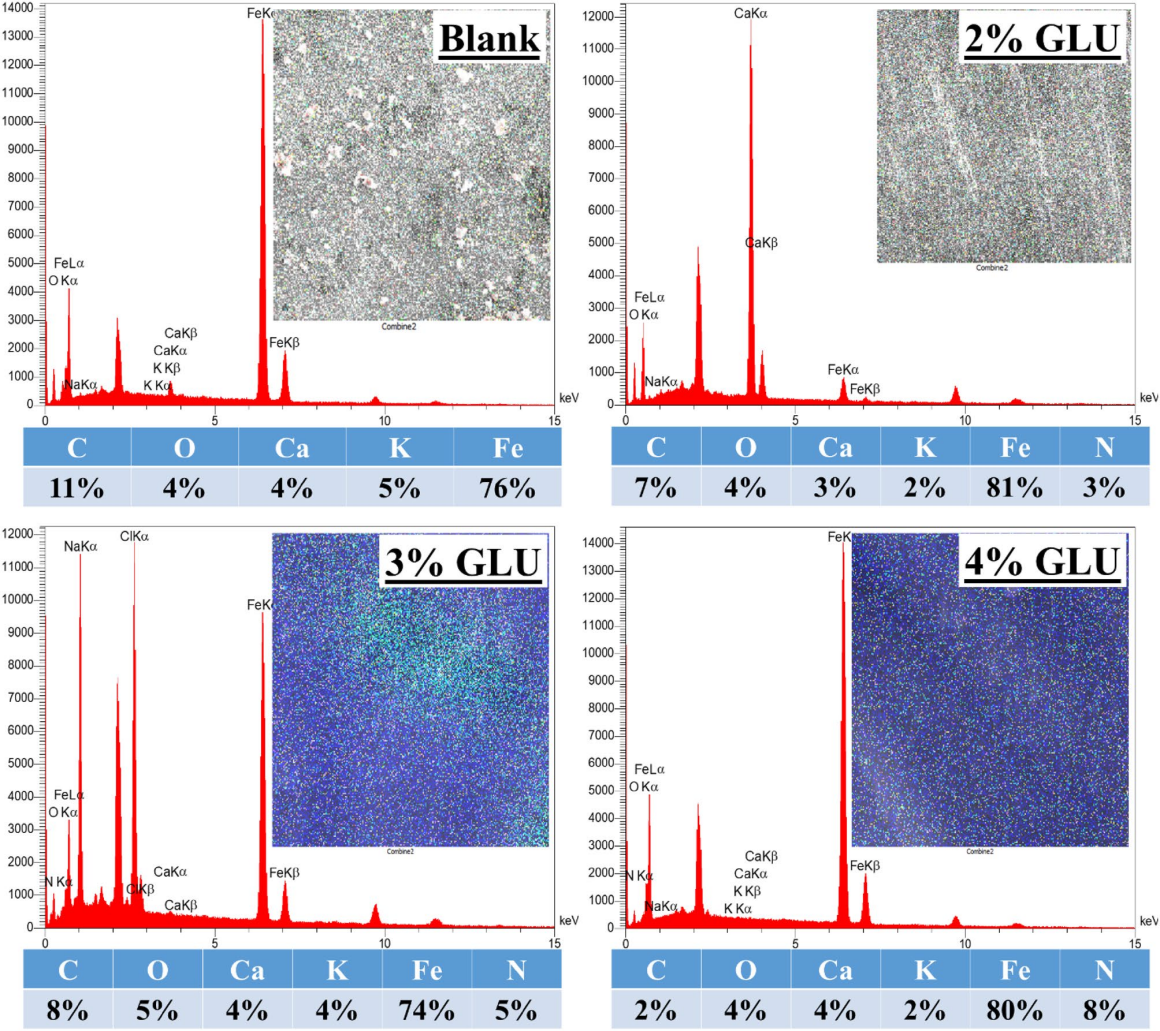
EDS/Mapping from the protected and unprotected samples after 48 h of immersion in CPS.

The elemental explorations evidenced that an O-based film covered the 48 h subjected blank sample that represents the passive layer and/or oxidized product coverage (Fig. 6). By introducing GLU into the simulated concrete pore solution for 48 h, the N-element was successfully detected over the protected sample surface, confirming that the GLU was adsorbed over the surface. Comparisons with the blank sample disclosed that the Fe content was raised alongside the O-element concertation reduction. The changes after protection directly correspond to the GLU layer development that limits the steel corrosion acceleration and corrosion products construction. Logically, by ascending the GLU concentration, the probability of N-element detection will enhance owing to a higher GLU molecules reaction with the immersed substrate surface.

### Contact angle

**Figure 7:**
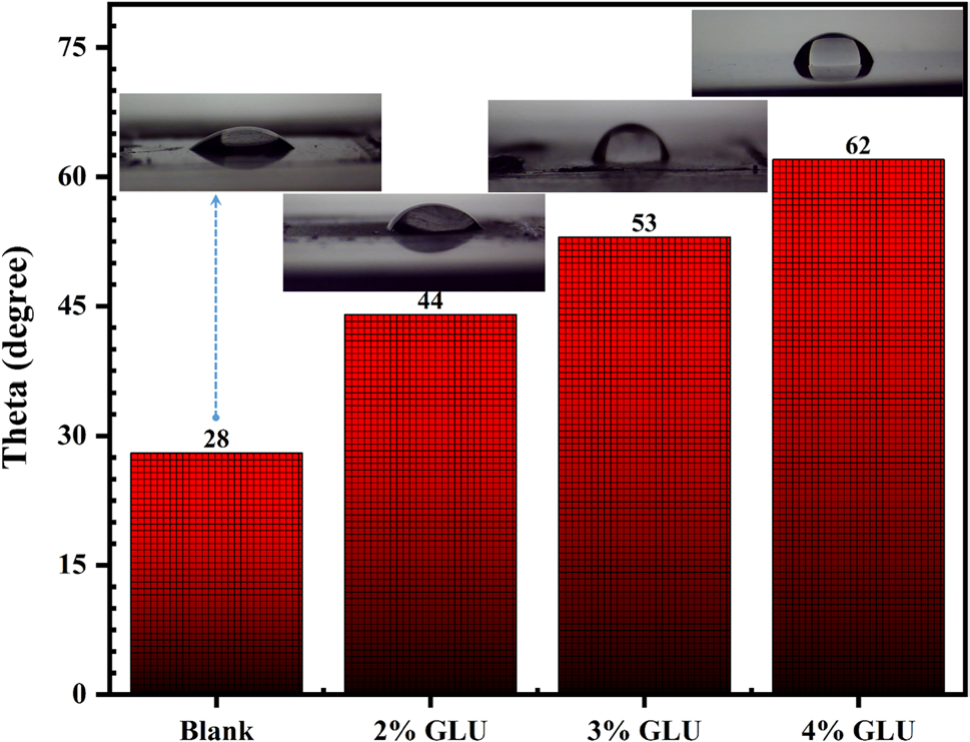
Contact angle captures from the protected and unprotected samples after 48 h of immersion in CPS.

The surface hydrophilicity provides remarkable information about the nature of the developed organic inhibitor film after 48 h of exposure. Clear glances evidenced that by increasing the inhibitor concentration, the surface hydrophobicity endured remarkable enhancement^34^ (Fig. 7). The surface hydrophobicity has a direct link with the existing hydrophobic groups in GLUs structure. By increasing the GLU concertation, a more hydrophobic layer will generate over the metal sample surface due to the GLUs inhibitive molecules coverage^35^. As a result, the contact angle increment occurred. However, the inhomogeneous structure of the corroded sample could promote the sample surface wettability and this is the reason for the noticeable reduction of the angle value of the blank sample.

### Raman spectroscopy results

**Figure 8:**
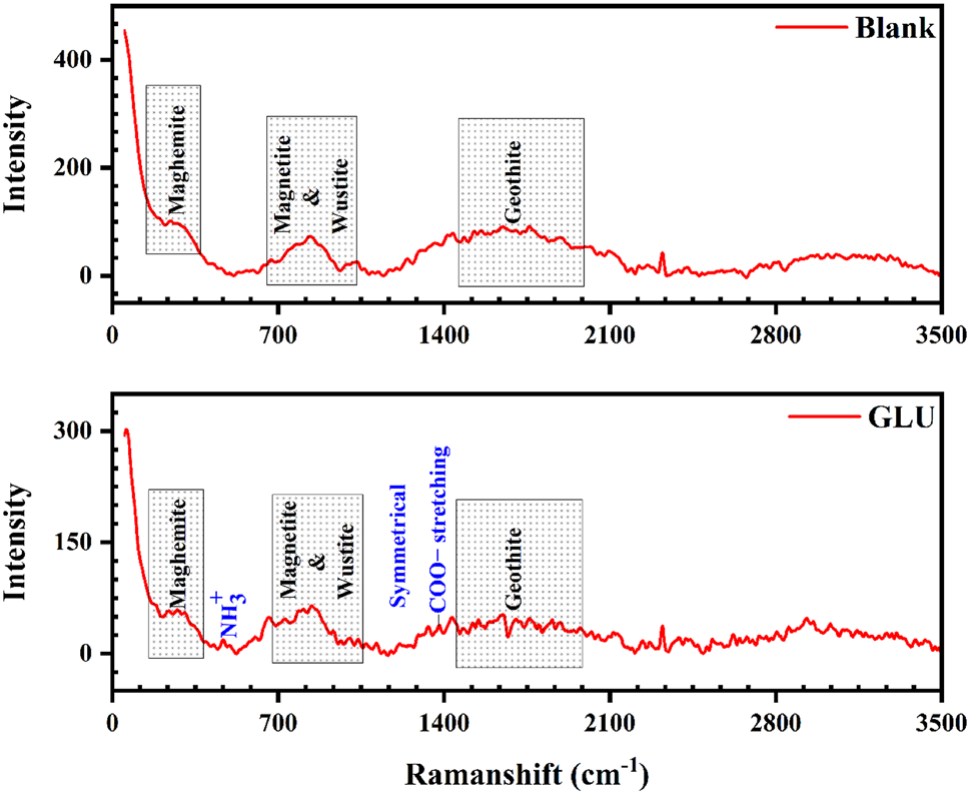
Raman analysis outcomes from the protected and unprotected samples immersed in CPS.

Raman test has been proposed by different literature as a non-destructive test to estimate the chemistry of the deposited film over the exposed sample. At first glance, it seems that both curves (blank and GLU) are similar. As explained in the “Inhibition mechanism” section, a passive oxidized layer will extend over the blank sample surface. The achievements clarified that the content of oxidized Fe products (including hematite (Fe_2_O_3_), maghemite (γ-Fe_2_O_3_), lepidocrocite (γ-FeOOH), goethite (α-FeOOH), magnetite (Fe_3_O_4_) and wustite (FeO) is extremely high. As a reason, the appearance of these peaks in the Raman spectra is quite logical (Fig. 8). These peaks were observed in the former studies too^36,37^.

Detailed explorations clarified that some peaks were detected from GLU protected samples, whereas they are unobservable in the blank sample spectrum. Near 500 cm^−1^ a small peak was detected which is corroborated by NH_3_^+^ twisting vibration. Also, the existence of a small hump around 1398 cm^−1^ represents the symmetrical COO^−^ stretching of GLU. These detections prove that the GLUs have been successfully adsorbed over the immersed steel surface^38^.

### GXRD results

**Figure 9:**
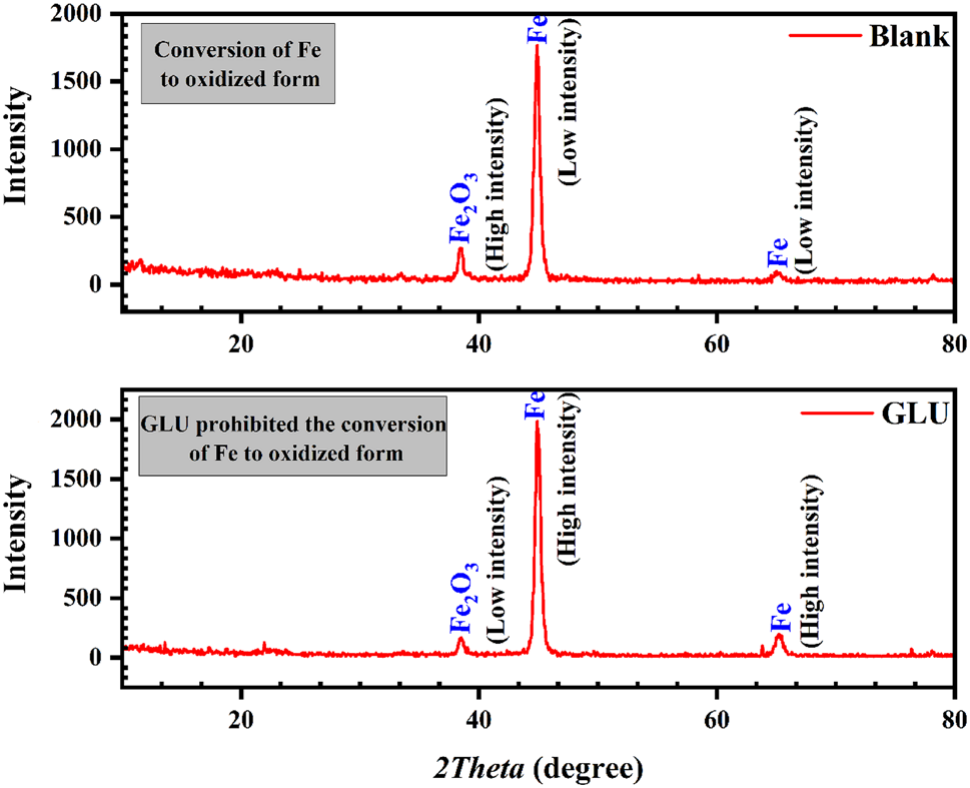
GXRD analysis outcomes from the protected and unprotected samples immersed in CPS.

For evidencing the chemical composition and estimating the crystal phase of the subjected samples, the GXRD diagrams were plotted. According to the PDF No.06-0696 library, the two characteristic peaks presented at 45 and 65° are pointing to pure Fe^39^ (Fig. 9). According to the literature, the peaks detected at 33 and 38° are related to the carbonated and hydroxide forms of calcium which are deposited over the substrate. Apparently, there are negligible differences between the blank and the protected samples' spectra, and in these circumstances, the comparison of peak intensity can notify promising information. Obviously, the Fe peaks of the blank sample have low intensity at both 45 and 65°. In other words, the rate of corrosion was substantially controlled in the presence of GLU and after adsorption, these molecules limited the conversion of Fe and loss of metal. Alongside, the Fe_2_O_3_ intensity was far higher than the sample protected by GLU. This can indicate that the GLUs successfully prohibited the Fe conversion to oxidized forms and Fe_2_O_3_ peak intensity will diminish as a result.

### Comparing results with previous works outcomes

Among all the listed studies in Table 3, due to acceptable performance besides affordability, organic inhibitors’ prevention impact has been compared with the present research. In most circumstances, researchers have gravitated toward anionic organic inhibitors to control the aggression rate of the chloride-contaminated CPS. The charged inhibitors can be easily chelated with the positively charged steel surfaces to obtain promising protection. For instance, in some recent works, Sodium lignosulfonate^40^, Polycarboxylates^11^, Alcamines^41^, Quaternium-32^33^, and Carboxylate derivatives^32^ were applied to protect the steel from corrosion against concrete pore solution attacks. All studies tried to survey the mentioned inhibitors' performance via different electrochemical and morphological methods and they claimed reliable results. Polarization studies have demonstrated that Lignosulfonate could overcome corrosion by anodic inhibition, while Polycarboxylates and alcamines displayed cathodic protection potency. Polarization explorations in the present work disclosed that like Lignosulfonate the GLU has slight anodic protection potency.

**Table 3:** Recent studies on corrosion inhibitors’ performance in CPS systems.

Inhibitor	*η* (%)	Immersion time (h)	CPS composition	Refrence
1,6-Hexanediamine (DIA) loaded Zeolite	96	336	0.02 M NaOH, 0.06 M KOH, 0.001 M Ca(OH)_2_ and 0.6 M NaCl	^42^
Sodium phosphate (Na_3_PO_4_), Sodium nitrite (NaNO_2_), and Benzotriazole (BTA)	BTA (97) > NaNO_2_ (72) > Na_3_PO_4_ (32)	936	Saturated Ca(OH)_2_ and 0.25 M NaCl	^16^
Calcium lignosulfonate (CLS)	98	10	Saturated Ca(OH)_2_ and 0.1 M NaCl	^43^
Polyacrylamide (PAM)	99	96	Saturated Ca(OH)_2_ and 0.01 M NaCl was added every 24 h	^44^
Imidazoline quaternary ammonium salt (IQS)	97	28	0.002 M NaOH, 0.0009 M KOH, 0.0004 M Ca(OH)_2_ and 0.6 M NaCl	^45^
Calcium lignosulfonate and Sodium oleate	97	720	Saturated Ca(OH)_2_ and 0.1 M NaCl	^10^
Triethanolammonium dodecylbenzene sulfonate (TDS)	90	16	0.2 M NaOH, 0.06 M KOH, 0.01 M Ca(OH)_2_, 0.24 M NaHCO_3_, and 0.25 M NaCl	^46^
Deoxyribonucleic acid (DNA)	58	168	Saturated Ca(OH)_2_ and 0.1 M NaCl	^12^
Polymethacrylate-co acrylamide	98	7	Saturated Ca(OH)_2_ and 0.6 M NaCl	^11^
TIPA85 and CA92 Alcamis	63 and 57	1	Saturated Ca(OH)_2_ and 0.6 M NaCl	^41^
Quaternium-32	93	0.5	Saturated Ca(OH)_2_ and 0.6 M NaCl	^33^
Sodium lignosulfonate	87	480	Saturated Ca(OH)_2_ and 0.08 M NaCl	^40^
Polycarboxylates	81	7	Saturated Ca(OH)_2_ and 0.6 M NaCl	^11^
Monosodium glutamate (GLU)	86	48	0.1 M NaOH, 0.4 M KOH, 0.1 M Ca(OH)_2_, 0.003 M CaSO_4_ and 0.6 M NaCl	Present work

Furthermore, EIS results revealed that GLU could control the steel corrosion in the intense chloride-contaminated CPS solution by 86% degree. Meanwhile, 0.0001 M Lignosulfonate purchased around 87% protection in a rather dilute Cl^−^ contaminated solution, and the inclusion of 7 wt% Polycarboxylate prohibited 81% of steel corrosion. Also, EIS measurement reflected that 150 ppm of TIPA85 and CA92 alcamines can provide 63% and 57% protection, respectively. All the reported results represent that the GLU as an organic inhibitor could have an outstanding inhibition performance for the steel bars of the reinforced concrete in a harsh corrosive media.

## Conclusion

- In this research, GLU was suggested as the promising potential inhibitor for tuning the anticorrosion index of the mild steel against CPS.
- EIS measurements clarified that 4 wt% GLU could vanish 86% of steel aggression and around 160,831 Ω cm^2^ total resistance was achieved.
- The polarization investigations disclosed an 80% corrosion inhibition degree and mixed inhibition mechanism of GLU.
- FE-SEM results demonstrated that GLU could limit steel corrosion prosperously by the formation of a uniform layer on the surface.
- The micrographs displayed that the corrosion products disappeared after the addition of the GLU inhibitor.
- Contact angle evaluations reflected a quasi-hydrophobic layer that could notably control the water spread on the surface.
- Raman and GXRD spectroscopic studies proved that the GLU-based layer controlled the anti-corrosion properties of the steel surface.

## Data Availability

All data generated or analyzed during this study are included in this published article.
